# Medical Education under Home Rule

**Published:** 1912-06-15

**Authors:** 


### The Hospital

The Workers' Newspaper of Administrative Medicine and Institutional Life, Administration, National Insurance and Health.

No. 1354, Vol. LII. SATURDAY, JUNE 15, 1912. PRICE ONE PENNY.

### MEDICAL EDUCATION UNDER HOME RULE.

In a brief paragraph last week we mentioned the resolution moved at the ninety-fifth session of the General Medical Council relating to the standards of medical and dental education under an Irish Parliament. Sir Charles Ball, it will be remembered, moved that "steps should be taken to procure the insertion in the Government of Ireland Bill of provisions reserving to the Imperial Parliament the control of legislation relating to the Medical and Dentists Acts." The matter is one of such supreme importance that it is to be hoped it may obtain consideration and discussion in circles which influence public opinion, with all due respect to the Medical Council, more than the deliberations of that body can honestly be said to do at the present time.

The problems attending the establishment of a separate Parliament and executive in Ireland are very numerous. Many of them are highly contentious and debatable, and are, of course, likely to be answered according to the political leanings and prejudices of the individual. But this matter of medical education is surely no party question at all, but one whose solution along equitable lines must appeal to everyone who has the well-being of future generations at heart; and that means every thinking man in every party, however great the differences may be between them as to the measures best calculated to promote the commonweal. Sir Charles Ball is an Irish surgeon whose reputation in the Empire has long been firmly established. Thus he was the only Irishman who contributed to Treves' "System of Surgery," published in 1895, which shows the estimation in which he was even then held by his professional colleagues. The incidents of his long and honourable career of forty years in his profession cannot be recited here; but anyone who will trouble to read up his record in the *Medical Directory* will easily be convinced that he is a man whose association with Irish medical education has been most intimate, and that his views upon its future are those of an expert indeed. In fact, he is probably better qualified to speak on this topic than any other individual in the still United Kingdom.

Sir Charles Ball pointed out, in support of the motion which he brought before the General Medical Council, that it would be within the competence of the Irish Parliament (as at present adumbrated) to make or amend the laws relating to medical practice. He thinks it possible that no lowering of the standard required for admission to the register would follow; but he feels sure that an agitation for such a lowering would be set on foot, and it is evident that he has a genuine fear lest such an agitation might be successful. The consequences of such an event would certainly be calamitous, not only to the people of Ireland but to those of Great Britain also. One effect might be the abolition of the reciprocity now existing between the constituent parts of the Kingdom; a step which would hit Irish graduates and licentiates far harder than British doctors. Such a step would raise many serious hardships, for the Imperial Services might be closed to Irishmen, and the position of those already practising in Britain or in the service of the Crown would be rendered very awkward and uncomfortable. This is only one of the calamitous results of any tampering with the present unified system of control of medical and dental education in these islands. There are many others, whose ramifications and consequences would be widespread and disastrous. The main fault of our system of medical education at present is that the element of central control is too weak, and that individual licensing bodies are allowed too much latitude in regard to their standards of qualification. Any proposals whose effect must be to increase this latitude and to diminish that control can only be denounced and resisted strenuously by all who have the future of medical efficiency at heart. Yet another ill-effect of any divergence of educational policies would be the difficulties in the way of the "one-portal" ideal which would be created.

It will be objected to this opinion that the prophecies of Sir Charles Ball are being taken too much for granted, and that no lowering of standards may follow the transfer of control from the Imperial to an Irish Parliament. The answer to this may not please either patriotic Nationalists nor Sir Charles Ball himself. It is that medical standards in Ireland are already dangerously lax, and that constant vigilance has been required to keep them from becoming lower still. The Irish graduate (or licentiate), as he is seen in Britain and in the Services, is an excellent fellow, with all the virtues typical of his race: cheerful, generous, chivalrous, warm-hearted, a loyal friend and nobody's foe. But, speaking broadly and of the mass, not of individuals (for there are, of course, many exceptions), his knowledge of medicine and surgery is not very profound, and his general level of professional excellence is below that of the average English or Scotch practitioner. It would be too much to expect Irish opinions to concur in this statement, but it is made in the fullest belief that it is true and that British medical opinion supports it: and this is why the mere possibility of any lowering of Irish standards fills us with the gravest alarm, and why we are inclined to agree with Sir Charles Ball in his attempt to render it impossible.

